# Computational interaction analysis of organophosphorus pesticides with different metabolic proteins in humans

**DOI:** 10.1016/S1674-8301(11)60045-6

**Published:** 2011-09

**Authors:** Amit Kumar Sharma, Karuna Gaur, Rajeev Kumar Tiwari, Mulayam Singh Gaur

**Affiliations:** a Pesticides Research & Sensors Laboratory, Department of Physics, Hindustan College of Science and Technology, Farah, Mathura-281122 (U.P.) India;; b Department of Bioscience, R. D. Govt. Girls College, Bharatpur, Rajasthan 321001, India.

**Keywords:** docking, organophosphorus pesticides, comparative analysis, modeling, toxicity analysis, acetylcholinesterases, *Homo sapiens*

## Abstract

Pesticides have the potential to leave harmful effects on humans, animals, other living organisms, and the environment. Several human metabolic proteins inhibited after exposure to organophosphorus pesticides absorbed through the skin, inhalation, eyes and oral mucosa, are most important targets for this interaction study. The crystal structure of five different proteins, PDBIDs: 3LII, 3NXU, 4GTU, 2XJ1 and 1YXA in *Homo sapiens* (*H. sapiens*), interact with organophosphorus pesticides at the molecular level. The 3-D structures were found to be of good quality and validated through PROCHECK, ERRAT and ProSA servers. The results show that the binding energy is maximum -45.21 relative units of cytochrome P450 protein with phosmet pesticide. In terms of H-bonding, methyl parathion and parathion with acetylcholinesterase protein, parathion, methylparathion and phosmet with protein kinase C show the highest interaction. We conclude that these organophosphorus pesticides are more toxic and inhibit enzymatic activity by interrupting the metabolic pathways in *H. sapiens*.

## INTRODUCTION

Pesticides, as a consequence of massive use in agriculture and other human activities, are widely distributed environmental contaminants and are subject to restricted use by legislations aimed at the protection of natural ecosystems and human health in India, Europe, the USA, and many other countries in the world. A wide structural variability also characterizes the pesticide subfamilies like insecticides, herbicides and fungicides, that are grouped together according to the target of biocide activity[1]. Pesticides, herbicides and insecticides have the potential to exert harmful effects on humans, animals, birds and other living organisms via inhalation or breathing, skin contact and food consumption. They also destroy our biodiversity and environment. Some possible changes occur after exposure such as skin irritations, nausea, breathing problems, cancer, hematological disorders, damage to the reproductive organs, and neurological disorders. The different routes of entry and the high binding efficiency of presticides with particular targets proteins in the human body have been taken into account.

Organophosphorus pesticides are the most frequently used pesticides in the world, with uses ranging from commercial and home use to agricultural applications for controlling unwanted insect pests[2]. Organophosphate pesticides include parathion, malathion, methylparathion, chlorpyrifos, diazinon, dichlorvos, phosmet, monocrotophos, fenthion, quinalphos, tetrachlorvinphos and azinphosmethyl. Organophosphorus pesticides obtain their toxicity from their ability to inhibit many metabolic and physiological enzymes like acetylcholinesterase (AchE), cytochrome P450, protein kinase C, and glutathione S-transferases (GSTs), causing neurotoxicity in humans[3]. For example, the presence of AchE in insects, birds, fish and all mammals give this class of pesticides enormous toxicity towards unintended targets to disrupt the endocrine, metabolic and digestive systems in the human body[3].

Human AchEs are a class of enzymes which catalyze the hydrolysis of acetylcholine (Ach), an ester which acts as a neurotransmitter. The reaction catalyzed by AchE is: Ach + H_2_O → choline + acetate[3]. The inhibition of AchE by organophosphorus pesticides occurs as a result of the phosphorylation of the serine residue in the active site. The hydroxyl group of serine residue acts as an electrophile which attacks the nucleophilic phosphorus. After phosphorylation, the protein is highly stable and the hydrolysis of Ach is blocked. In some cases, depending on the chemical structure of the pesticide, the phosphorylation and inhibition may be irreversible[3]. The inhibition of the protein causes formation of Ach in the neural synapses. The main function of AchE is to recycle Ach by its hydrolysis at cholinergic synapses in order to restore the membrane potential after propagation of a nerve impulse[3]. The principle of cholinesterase inhibition i.e. in a biosensor is used as a means of detection of a phosphorothionate ester compound and inhibition of AchE may be low or absent[4]. It is reported that AchE is a target enzyme for biologically active compounds ranging from anti-Alzheimer disease agents acting as reversible inhibitors to organophosphorus pesticides[5] and warfare agents which act as reversible or irreversible inhibitors.

The activation of organophosphate pesticides has been attributed to the cytochrome P450 family of enzymes. P450s have also been shown to carry out direct detoxification of organophosphorus pesticides through dearylation. Humans have the ability to activate organophosphorus pesticides through CYP1A2, 2B6, 2C19, and 3A4 cytochromes, which are particularly sensitive to their actions[6]. This hypothesis reported that the activation and detoxification of parathion and chlorpyrifos pesticides are found in human liver microsomes[7]. In addition, the recombinant human P450 protein was used to quantify organophosphorus pesticides by human liver microsomes and the kinetic values may vary widely because of the marked variability of P450 content and activity in procured human specimens and differences in incubation conditions and analytical methods[8].

The third protein which we target are GSTs, a widely distributed family of detoxifying dimeric enzymes found in most forms of life. GSTs inactivate toxic effects by chemically bonding them to the tripeptide glutathione, making them soluble so that the body can easily excrete them. Pathogenic parasites also make their own GSTs to help them inactivate the drugs[9]. Resistance to organophosphorus pesticides is considered to be due to the metabolism of these compounds by GSTs[10]. Many researchers state that insecticide resistant insects have elevated levels of GST activity in crude homogenates, which suggests a role for GSTs in resistance[10]. Multiple forms of these enzymes have been reported for mosquitoes, house fly, Drosophila, sheep blow fly and grass grub[11]. GSTs are also involved in intracellular transport, biosynthesis of hormones and protection against oxidative stress. In addition, they contribute to the removal of toxic oxygen free radical species produced through the action of pesticides. They have peroxidase[12] and isomerase activity[13], which inhibits the junction of N-terminal kinase (thus protecting cells against H_2_O_2_-induced cell death) and they are able to non-catalytically bind a wide range of endogenous and exogenous ligands[14].

The fourth protein, i.e. protein kinase C (PKC), is also one of the most key target proteins for organophosphate pesticide toxicity, because it can also have an effect on signaling pathways by activating PKC[15]. PKC may mediate signaling effects and to date there has been no information regarding whether these xenobiotics can modulate PKC, which is a significant event signaling the increase in endothelial permeability and cell proliferation. However, the activation is probably indirect through organophosphorus pesticides-mediated formation of reactive oxygen species (ROS) that activate PKC, which can be measured in human liver and brain cytosolic fractions[15]. Organophosphorus pesticides will induce ROS formation and oxidative stress has also been shown to be associated with apoptosis in different tissues. Organophosphorus pesticides can also inhibit the steroid androgen receptor (AR), which causes steroid hormone disturbances in the human body. It is reported that in hepatic tissues, the greatest increase in activities was observed with TCDD (2, 3, 7, 8-tetrachlorodibenzo-p-dioxin; herbicide), chlorpyrifos, endrin and Cd (II), while chlorpyrifos and fenthion exerted the greatest increases in the brain tissues[15]. Protein kinase C may be a best target protein of free radicals and oxidative stress, leading to altered cell proliferation and differentiation[15].

The last protein is α1-antichymotrypsin (ACT), an inhibitor of proteinases of the chymotrypsin class. It is also activated in Alzheimer's disease patients. ACT is synthesized primarily in hepatocytes and secreted into the blood[16]. The expression of ACT in hepatic cells is known to be enhanced by interleukin-6 (IL-6), to some extent by IL-1, and also by glucocorticoids[17]. ACT is synthesized in human bronchial and breast epithelial cells, epididymal cells, predominantly in the choroid plexus in normal brain, and in astrocytes or astroglia, and to a small extent in monocytes[16]. Brain microvessel endothelial cells also release ACT[17], indicating that the inhibitor may also perform unique functions in local microenvironments. ACT may be also involved in controlling the oxidative damage because of its correlation to inhibition of oxygen consumption and superoxide generation in human granulocytes. Complexes formed by ACT and chymotrypsin regulate the production of superoxides in neutrophil membranes by interacting with NADHP oxidase[18].

Molecular docking is a frequently used method in computer-aided drug design. It evaluates how small molecules called ligands like organophosphate pesticides and the target macromolecules (e.g. receptor, enzyme or nucleic acid) fit together. Hence, this research hypothesis focuses on a comparison and interaction study of five different metabolic and physiological proteins [PDBIDs: 3LII, 3NXU, 4GTU, 2XJ1 and 1YXA in *Homo sapiens* (*H. sapiens*)], which are inhibited by the exposure to ten different organophosphorus pesticides and exert their toxic effects on humans metabolism, by using online bioinformatics tools and softwares. We are paying attention to the interaction of organophosphorus pesticides with target proteins and predict which one is more toxic and reveal the injurious effects on human by dermal layers exposure, inhalation, eyes exposure and oral contact.

## MATERIALS AND METHODS

### Model description

The crystal structures of five different proteins, AchE, cytochrome P450, GST, PKC and ACT, were obtained from the Protein Data Bank (PDB) website (http://www.rcsb.org/) in *.pdb (dot pdb) format[19]. This format is recommended for protein structures that are obtained by X-ray crystallography or NMR studies. For further analysis, we selected recombinant human AchE (PDBID: 3LII)[20], which has a 3.20 Å resolution. The second one was the crystal structure of human cytochrome P450 3A4 bound to an inhibitor ritonavir (PDBID: 3NXU)[21] and it has a 2.0 Å resolution. The third was ligand-free homo dimeric human GST m4-4 (PDBID: 4GTU)[22], which has a 3.30 Å resolution. The protein kinase pim-1 in complex with a small molecule inhibitor (PDBID: 2XJ1)[23] has a 2.13 Å resolution and serpina3n, a murine orthologue of human α1-antichymotrypsin (PDBID: 1YXA)[24] has a 2.10 Å resolution. Crystallographic waters and hetero atoms were removed and the structures were fully solvated before docking analysis.

### Sequence alignment

AchE, P450, GST, PKC and ACT are composed of 534, 457, 217, 273, and 372 amino acids, respectively. The protein sequence files were obtained from the PDB in *.txt (dot txt) format. Multiple alignments of the related sequences were performed using the ClustalW program accessible through the European Bioinformatics Institute website (http://www.ebi.ac.uk/Tools/clustalw2/index.html)[25].

### Secondary structure prediction

Secondary structure analyses of AchE, P450, GST, PKC and ACT in humans were described in Protein Data Bank. Prediction of different domains of all these proteins was carried out through the SUPERFAMILY sequence search server[26] by using the hidden Markov models.

### Model validation

The validation of AchE, P450, GST, PKC and ACT protein structures were assessed by using Ramachandran plot through PROCHECK validation package[27]. The initial model showed problems in the conformation of different loop regions, which were subjected for loop modeling and further validated by ERRAT web server[28]. ERRAT plot gives measurement of the structural error for each residue in the 3-D structure model. This process was repeated iteratively until most of the amino acid residues were below 95% cut-off value and the residues which lie above 95% cut-off value were subjected to loop modeling in MODELLER. Finally, all protein models showing the best PROCHECK and ERRAT plot were subjected to native protein folding energy evaluation by using ProSA program[29]. ProSA is an interactive web which requires the atomic coordinates of the model to be evaluated and recognition of errors in three-dimensional structure.

### Protein function analysis

We also analyzed the novel functions of AchE, P450, GST, PKC and ACT proteins by means of SVMProt server (BIDD server) with the aim of Support Vector Machine (SVM) learning techniques, which classify proteins into functional families from its primary sequences[30].

### Ligand binding site (active site) prediction

Pocket-Finder or Q-site finder is a molecule-binding site prediction server based on the Ligsite algorithm and also compares with the CASTp server[31],[32]. It works by scanning a probe radius 1.6 Å along all gridlines at a grid resolution of 0.9 Å surrounding the protein. The probe also scans cubic diagonals. Grid points are defined to be part of a site when the probe is within range of protein atoms followed by free space followed by protein atoms. Grid points are only retained if they are defined to be part of a site at least five times.

### Protein–ligand interaction study (docking)

The 3-D structures of AchE, P450, GST, PKC and ACT were further used for insilco docking study to know the interaction between the organophosphorus pesticides and target proteins. Various methods applied in this study are given below

#### Preparation of proteins

The crystal structures of AchE, P450, GST, PKC, and ACT and their PDBID, 3LII, 3NXU, 4GTU, 2XJ1, and 1YXA[20]–[24] were downloaded from the PDB in *.pdb (dot pdb) format. The crystal structures of proteins have already presented some ligand molecules, which were removed. The other necessary action was to remove water molecules from the surface of the protein molecules. It is compulsory because the extra water molecules will mask the protein surface from the ligand. All protein 3-D models were prepared for docking by removing waters and hetero atoms for docking analysis by using PyMol V2.0.7 software[33].

#### Preparation of ligand

The three dimensional structures of ten organophosphate pesticides were downloaded from the Pubchem compound search of NCBI database[34]. The structures were downloaded as *.sdf (dot sdf) file format. The 3-D structure of ten organophosphate pesticides like malathion, parathion, methylparathion, monocrotophos, chlorpyrifos, fenthion, quinalphos, phosmet, tetrachlorvinphos and azinphosmethyl have Pubchem IDs are: CID4004, CID991, CID4130, CID5371562, CID2730, CID3346, CID26124, CID12901, CID5284462 and CID2268, respectively. The 3-D structures of organophosphate pesticides were open through PyMol V2.0.7[33], a visualization tool and converted into *.pdb file.

#### Docking studies using PatchDock and FireDock

The crystal structures of AchE, P450, GST, PKC and ACT were used for docking analysis through PatchDock (http://bioinfo3d.cs.tau.ac.il/), where candidate solutions were generated by rigid-body docking methods[35]. PatchDock determined the best starting candidate solutions based on shape complementarily of soft molecular surfaces of proteins. The Clustering RMSD was 4.0 Å for analysis and complex type was set to as default. The PatchDock algorithm divides the Connolly dot surface representation of the molecules into concave, convex and flat patches. Then, complementary patches are matched in order to generate candidate transformations[35]. Each candidate transformation is further evaluated by scoring function that considers both geometric fit and atomic desolvation energy. The 1000 best docked candidate transforms from PatchDock, based on global energy, attractive and repulsive van der Wall's interactions, partial electrostatics, atomic contact energy (ACE), and additional estimations of the binding free energy were used in FireDock (http://bioinfo3d.cs.tau.ac.il/)[36]. FireDock re-scored the 10 top candidate solutions by restricting the flexibility to the side-chains of the interacting surface and allowing small rigid-body movements. This study was carried out by selecting the first best candidate solution from FireDock were retained and then visualized through PyMol V2.0.7[33].

## RESULTS

### Model description

The length of amino acid residues of AchE, P450, GST, PKC and ACT varies between 217 and 534 amino acids. The downloaded structures are described as text files, which contain necessary information on the molecule such as the number of atoms, name of atoms, bond distances, angles, dihedral angles, and the number of residues. Multiple sequence alignments of five amino acid sequences were carried out through the ClustalW program, which showed that sequences were not identical to each other.

**Fig. 1:**
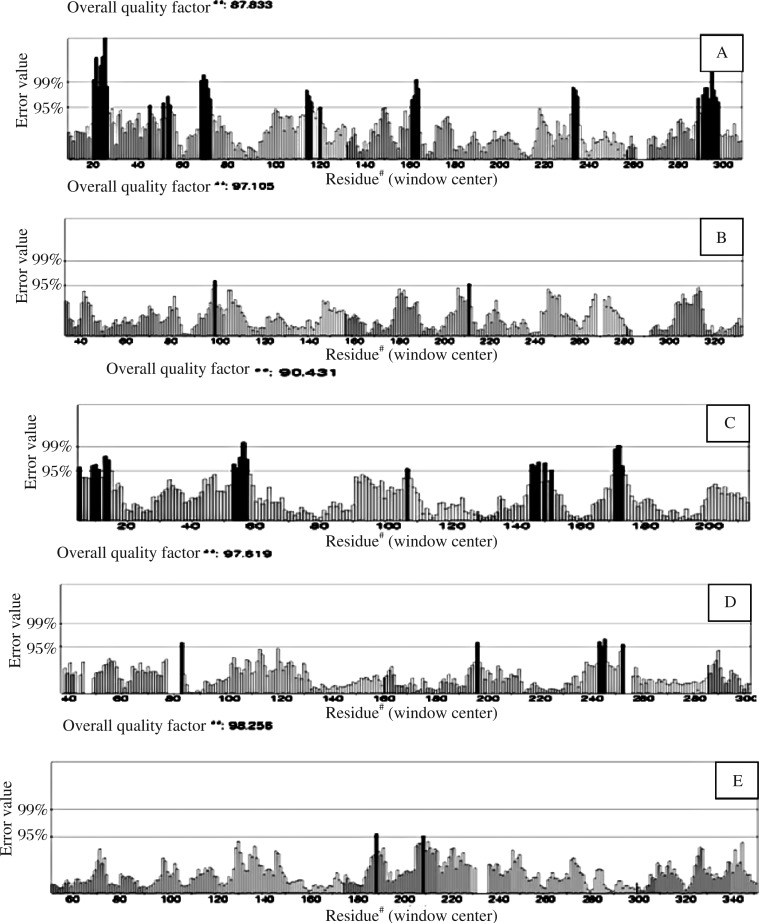
The overall quality score (E-value) of A, B, C, D, E represents AchE, P450, GST, PKC and ACT proteins respectively by using ERRAT server.

The three dimensional model of five proteins in *H. sapiens* were validated by VERIFY 3-D score predicted by the SAVES server[37]. VERIFY 3-D analyzes the compatibility of an atomic model (3-D) with its own amino acid sequence. For all 3-D models, the range varies between 94.10% and 98.54% for ACT and PKC, respectively. The residues have a score of greater than 0.2, which indicates a good quality model (***Table 1***). The ERRAT server is used for analyzing the statistics of non-bonded interactions between different atom types and scores greater than 50 are normally acceptable. For all five protein models, ERRAT score varies between 98.256 and 87.833 of 1YXA (ACT) and 3LII (AchE), respectively, which fall within normal range for high quality models (***Fig. 1*** and ***Table 1***). ProSA is widely used to check 3-D models of protein structures for potential errors. The Z-score indicates overall model quality and measures the deviation of the total energy of structure with respect to an energy distribution derived from random conformations. The ProSA score was negative for the modeled protein, which indicates its validity. The ProSA profiles calculated the protein structures, which were found similar to the energy of all five different structures of protein from PDB listed in ***Table 1***. The overall model quality of all five 3-D structures was reflected by the minimum Z-score, which was -7.63 for PKC (PDBID: 2XJ1) and the maximum Z-score, which was -10.62 for AchE (PDBID: 3LII) as is shown in ***Table 1***.

**Table 1:** Ramachandran map of AChE, P450, GST, PKC and ACT proteins there PDBIDs are 3LII, 3NXU, 4GTU, 2XJ1 and 1YXA respectively were calculated with the PROCHECK program

Model Description	3LII	3NXU	4GTU	2XJ1	1YXA
Residues in most favoured regions [A,B,L]	358/82.1%	356/90.4%	217/91.4%	224/94.1%	308/91.7%
Residues in additional allowed regions [a,b,l,p]	71/16.3%	36/9.1%	8/4.0%	12/5.0%	28/8.3%
Residues in generously allowed regions [∼a,∼b,∼l,∼p]	4/0.9%	1/0.3%	1/0.6%	1/0.4%	0/0.0%
Residues in disallowed regions	3/0.7%	1/0.3%	3/0.7%	1/0.4%	0/0.0%
ERRAT overall quality score	87.833	97.105	90.431	97.619	98.256
VERIFY 3-D (3D-1D score > 0.2)	97.76%	96.29%	95.54%	98.54%	94.10%
ProSA Z-score	-10.62	-9.84	-8.93	-7.63	-8.64
SCOP (Domains)	Region: 3-530	Region: 5-457	Region: 85-216	Region: 4-263	Region: 1-371
			Region: 2-84		

The Z-score and ERRAT overall quality score by servers like ERRAT and SUPERFAMILY sequence search server.

### Model validation

The geometry of AchE, P450, GST, PKC and ACT 3-D structures were evaluated through Ramachandran plot calculations by using PROCHECK. Stereochemical evaluation of backbone Psi (Ψ) and Phi (Φ) dihedral angles of five human proteins were revealed in different percentages i.e. 82.1%-94.1%, 4.0%-16.3% and 1%-4% residues were diminishing within the most favored regions, additionally allowed regions and generously allowed regions, respectively (***Table 1***). The dihedral angles revealed that some residues like 1%-3% disallowed regions of Ramachandran plot. The model has normal distribution of residue types over the inside and the outside of the protein structures. The residues in the disallowed region were ignored as they were not present near the active site nor were they involved in ligand binding.

By using the SUPERFAMILY sequence search server, the crystal structure of AchE consists of one interacting domain that belongs to the family of AchE, an alpha/beta-hydrolase N-terminal domain (residues 5-530) and their E-value is 4.34e-163. The cytochrome P450 (3NXU) has one domain that lies between the 5 and 457 amino acids and belongs to the cytochrome P450 superfamily and the E-value is 1.83e-135. The third one is GST (4GTU), which has two domains that lie between region 85-216 and 2-84 and their superfamily are GST C-terminal domain-like and thioredoxin and the expected E-value was 1.77e-46 and 1.67e-23, respectively. Similarly, in the fourth and fifth proteins, PKC (2XJ1) and ACT (1YXA) have one family that lies between amino acids 4-263 and 1-371 and belongs to protein kinase-like (PK-like) and serpins superfamily, and their E-value was 1.25e-71 and 2.23e-135, respectively, that is assigned by SCOP domains.

### Prediction of secondary structures

**Fig. 2:**
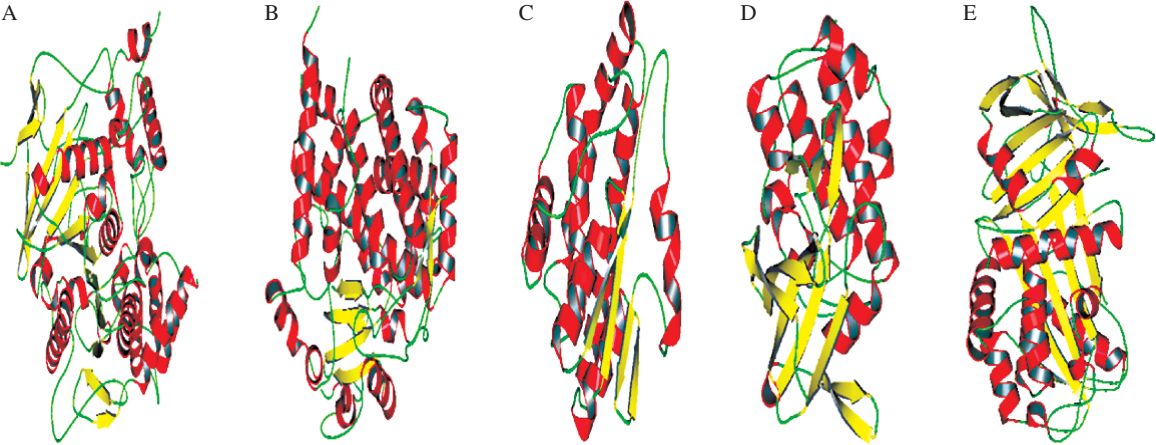
Secondary structure of five different human proteins. A: human acetylcholinesterase (3LII). B: cytochromeP450 (3NXU). C: gutathione S-transferase (4GTU). D: represents protein kinase C (2XJ1). E: is alpha-1-antichymotrypsin (1YXA). The arrow indicates the β-sheets with flexible residues shown in yellow, α-helices in red color and green are the loop regions. The protein structures were visualized through PyMol V2.0.7.

The secondary structure prediction, which was updated in PDB used in the analysis, revealed that random coils dominated among secondary structure elements followed by alpha helix, extended strand and beta turns. The crystal structure of AchE, P450, GST, PKC and ACT consists of a total of 8 to 21 α-helices in GST and P450 proteins and the number of β-sheets varies between 4-16 in GST and AchE, respectively, as is shown in ***Fig. 2***. The N-terminal domain consists of two anti-parallel β-sheets, forwarding two β barrel domains in 3LII and 3NXU crystal structure. The human AchE protein (3LII), which consists of 16 mixed β-sheets and 20 large and small α-helices, similarly in P450 (3NXU), has one anti-parallel β-sheet, seven mixed β-sheets and 21 α-helices. The third one is GST (4GTU), which has 2 pairs of anti-parallel β-sheets and eight α-helices, and the fourth is the PKC (2XJ1), which has 12 mixed β-sheets and 13 α-helices. The last is ACT (1YXA), which contains 14 β-sandwiches and 12 α-helices (***Fig. 2***).

### Functional analysis by support vector machine

**Fig. 3:**
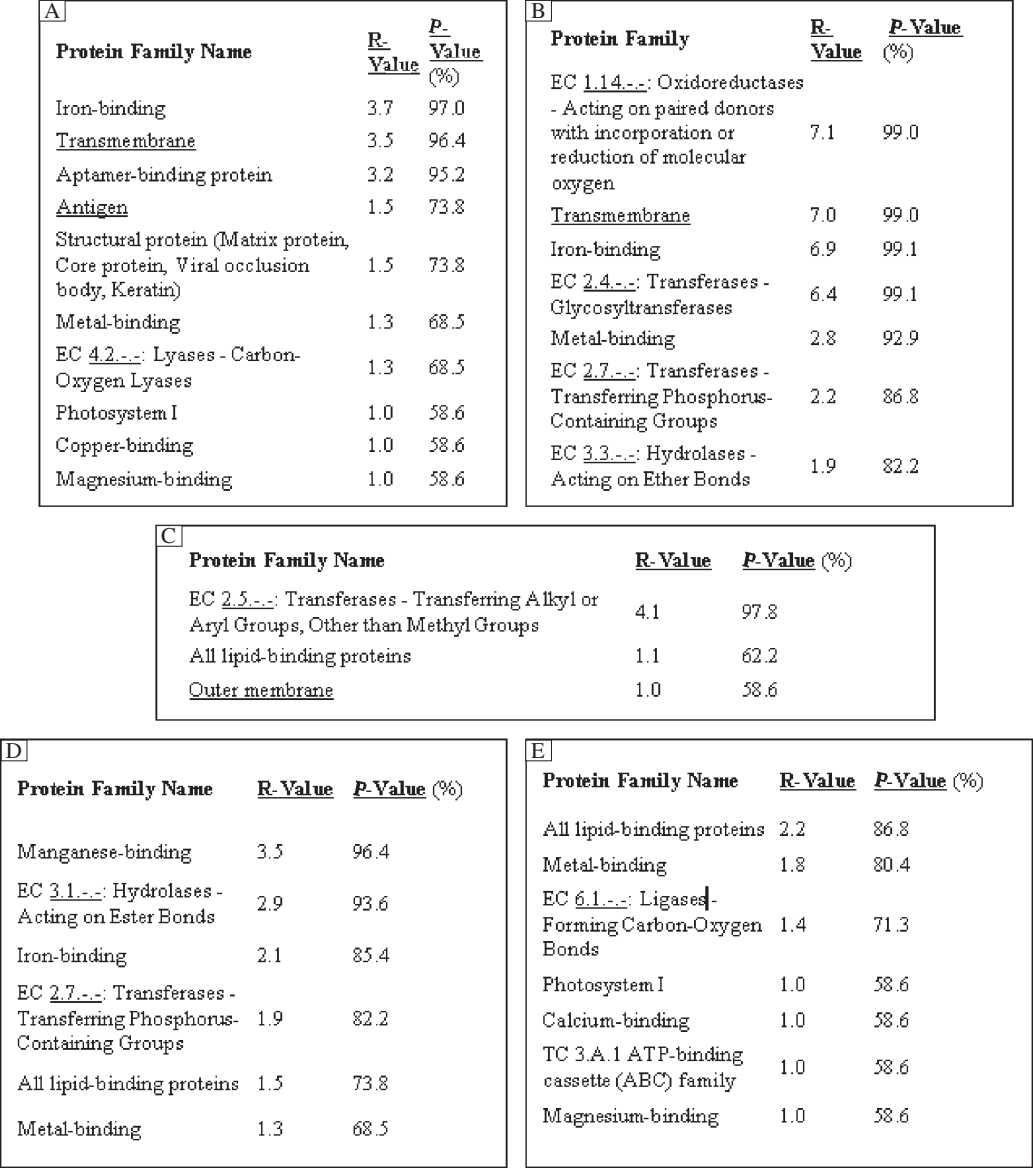
Comparative analysis of functional assignment of five different human proteins by SVMProt method through BIDD server. A: acetylcholinesterase. B: cytochrome P450. C: gutathione S-transferase. D: protein kinase C. E: alpha-1-antichymotrypsin proteins.

Different unknown and hidden functions of five different human proteins were predicted using machine learning technique like a statistical support vector machine-based classifier i.e. SVMProt (***Fig. 3***). The comparative analysis of AchE, P450, GST, PKC and ACT for functional assignment shows that it belongs to the transferase group of proteins as shown in ***Fig. 3***. AchE (3LII) and P450 (3NXU) belong to the transmembrane region proteins. Human proteins like AchE, PKC and α1-antichymotrypsin have magnesium binding (58.6%), metal-binding properties. AchE and P450 have the tendency to bind with iron (97.0% and 99.1%) and also copper (58.6%) properties, respectively. Comparative analyses have shown that different domains of α1-antichymotrypsin belong to ATP-binding cassette (ABC) function shown in ***Fig. 3***.

### Ligand binding site analysis

**Fig. 4:**
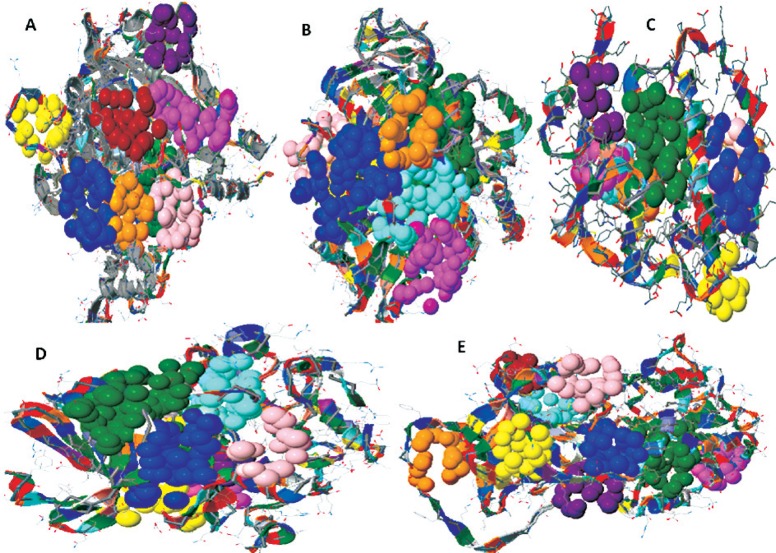
The possible binding sites which were obtained by using CASTp server of all five proteins in human. A: human acetylcholinesterase (3LII). B: cytochrome P450 (3NXU). C: gutathione S-transferase (4GTU). D: protein kinase C (2XJ1). E: α-1-antichymotrypsin (1YXA). The possible binding site indicates in various colors like blue, green, cyan, etc.

The potential ligand binding sites (LBSs) of AchE, P450, GST, PKC and ACT were identified by Pocket Finder program. A total of ten possible binding sites were obtained in all five proteins. The possible active sites obtained from the CASTp server in all five proteins are shown in ***Fig. 4***. The frequently involved amino acid residues in human AchE are Gln71, Tyr72, Asp74, Glu81, Thr 83, Tyr124, Glu202, Ser203, Trp236, Tyr337, Pro368, Arg463 and Asn533. The involved amino acid residues in P450 proteins in forming the pocket are Ile50, Tyr53, Arg105, Arg106, Pro107, Gly109, Glu144, Arg212, Phe213, Glu374, and Ile443 (***Table 2***). Similarly, in the case of GST, amino acid residues involved in different active sites are Tyr6, Trp7, Ile9, Arg10, Arg42, Met104, Asn108, Leu110, Tyr115, Asp161, Lys207 and Tyr208. The frequently involved amino acid residues in various active sites of PKC of *H. sapiens* are Leu44, Arg73, Ile74, Ser75, Asp76, Arg122, Glu171, Ile185, Gly188, Ser189 and Asp202. The last protein for analysis is an α1-antichymotrypsin which has involved residues in different active sites, including Asp68, Ser77, Arg123, Asn125, Val131, Thr135, Gly136, Gln160, Tyr181, Gln185, Tyr208, Met282, Pro395, and Lys413. The top five different active sites predicted through Pocket Finder in five different proteins like AchE, P450, GST, PKC and ACT are listed in ***Table 2***.

### Protein-ligand interaction analysis

**Fig. 5:**
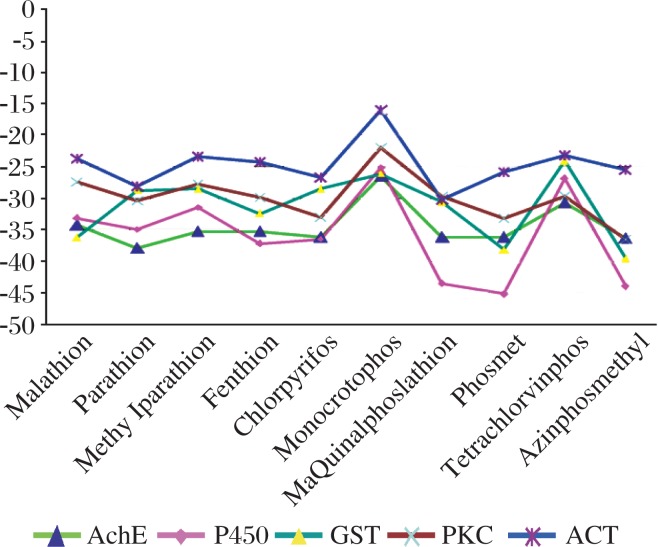
The graphical plot shows the global energy i.e. dock score of five different metabolic proteins i.e. acetylcholinesterase (AchE), cytochrome P450 (P450), glutathione S-transferases (GST), protein kinase C (PCK) and α-1-antichymotrypsin (ACT) with ten different organophosphorous pesticides.

**Fig. 6:**
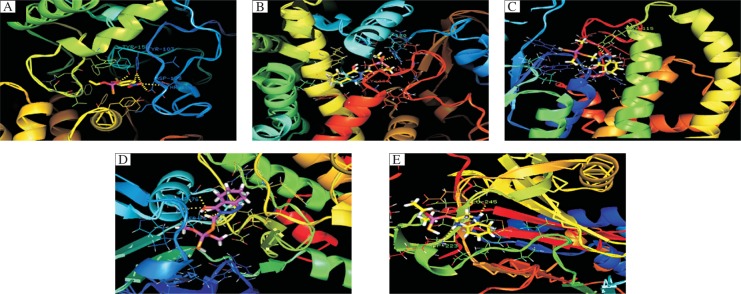
Molecular docking through PatchDock and refined by FireDock. A: It represent the acetylcholinesterase (Pdbid: 3LII) with parathion pesticides and dock score is -37.90. B: The highest binding energy is -45.21 of cytochrome P450 protein (Pdbid: 3NXU) with phosmet. C: Similarly, the glutathione S-tranferase (Pdbid: 4GTU) with ligand as an azinphosmethyl pesticides and score is -39.60. D: Binding energy -36.57 of protein kinase C (Pdbid: 2XJ1) with azinphosmethyl. E: Alpha-1-antichymotrypsin (Pdbid: 1YXA) with quinalphos are -30.15. Structures were visualized using PyMol V2.0.7 software viewer.

Docking represents the mathematical calculation of the most probable spatial orientation of two interacting molecules, usually protein and small ligand, two interacting proteins or DNA and protein. Various parameters are calculated to evaluate possibility of such protein-ligand interaction. For molecular docking analysis, we used new server PatchDock and refinement tool FireDock[35]. It has previously been proved that all organophosphorus pesticides have toxic effects on mammals, birds, fish, reptiles and insects[3]. But there is no report about the interaction of these ten organophosphorus pesticides with target proteins that are involved in the human metabolic and digestive pathway till date. We accessed the tertiary structures from PDB with the proposed interaction of five different human proteins like AchE (3LII), cytochrome P450 (3NXU), GST (4GTU), PKC (2XJ1) and α1-antichymotrypsin (1YXA) with these organophosphorus pesticides by using these tools. They are summarized in ***Fig. 5*** and ***Table 3***. The docking solution was visualized using the program PyMol V2.0.7 software viewer and distance measurements were carried out with the same software package. Global energy function (i.e. dock score) of five proteins with their highest interaction energy with organophosphorus pesticides are calculated by the FireDock server to be -45.21 relative units (this value is considered to be related to free binding energy and higher negative value means higher free binding energy and thus higher interaction probability). It is interesting to note that organophosphorus pesticide phosmet has shown maximum ligand protein interaction (dock score: -45.21) with human P450 protein in which frequent involvement of H-bonds (4) and of both hydrophobic and basic amino acid residues including Arg130, Ile443, and Gly444 (***Table 4*** and ***Fig. 6B***). The second highest dock score is -39.60 for GST (Pdbid: 4GTU) with an azinphosmethyl pesticide as a ligand and the involved hydrophobic amino acids, Tyr115 and Tyr6, are shown in ***Fig. 6C***. AchE bound to parathion shows the third highest binding energy, which is -37.90 (***Table 4*** and ***Fig. 6A***), and their involved amino acids residues are Tyr155, Tyr103, Asp105 and Thr106, which also belong to the active site residues predicted through pocket finder and the CASTp server (***Table 4*** and ***Fig. 4***). Similarly, the fourth and fifth highest binding energy is PKC bound to azinphosmethyl and ACT bound to quinalphos, which is -36.57 and -30.15, respectively, and the number of H-bonding is 4 and 2, respectively.

In the case of AchE, the binding energy in descending order with organophosphorus pesticides, which exert toxic effects on the human body, varies in between -37.90 to -26.53 for parathion, chlorpyrifos, azinphosmethyl, phosmet, quinalphos, fenthion, methylparathion, malathion and monocrotophos and is shown in ***Fig. 5***. Interaction with the above mentioned pesticides, the frequent involvement of H-bonds varies between 1 and 6 and the highest interaction of AchE was found to be with methylparathion (6) followed by parathion (5), malathion and monocrotophos (4). The amino acids of AchE forming hydrogen bond interaction with methylparathion are Trp182, Arg13, and Asn186, and with parathion are Tyr155, Tyr103, Asp105, Thr106, Ser125 and Ser234, which suggests that these pesticides interact with AchE (***Fig. 6A***) and have inhibitory action which occurs as a result of the phosphorylation of the serine residue in the active site of the enzyme.

**Table 2:** The top five involved amino acids residues in active sites in five different human proteins by using Pocket Finder server.

AchE	P450	GST	PKC	ACT
SITE:1	SITE:1	SITE:1	SITE:1	SITE:1
Gln71,Tyr72	Ile50, Tyr53, Phe57, Asp76,	Tyr6, Trp7, Ile9,	Leu44, Gly45,	Ser77, Ala80,
Asp74, Thr 83	Gln79, Arg105, Arg106, Pro107,	Arg10, Gly11,	Gly48, Phe49,	Leu84, Leu121,
Trp86, Asn87	Phe108, Gly109, Ile118, Ser119,	Leu12, Arg42,	LYS67, Val69,	Asn125, Thr135,
Pro88, Glyl20, Glyl21,	Ile120, Trp126, Thr136, Phe137,	Ala103, Met 104,	Arg73, Ile74,	Gly136, Ser137,
Glyl22, Tyrl24, Glu202,	Glu144, Met145, Thr187, Ser188,	Ser107, Asn108,	Ser75, Asp76,	Ala138, Tyr159,
Ser203, Trp236, Trp286,	Lys208, Leu210, Leu211, Arg212,	Leu110, Ala111,	Glu89, Le104,	Gln160, Tyr181,
Leu289, Ser293,Val294	Phe213, Ile223, Thr224, Phe241,	Tyr115, Leu158,	Leu120, Arg122,	Gln185, Val206,
Phe295, Phe297	Arg268, Asp270, Ile301, Phe302,	Asp161, Leu165,	Leu174, Glu171,	Asn207, Tyr208
Tyr337, Phe338	Phe304, Gly306, Glu308, Thr309,	His166, Lys207,	Ile185, Gly188,	
Tyr341, His447, Gly448,	Thr310, Arg372,	Tyr208, Thr209	Ser189, Asp202	SITE:2
Ile451	Leu373, Glu374,			Arg123, Leu124,
	Cys442,Gly444	SITE:2	SITE:2	Lys128, Asp129,
SITE:2	Phe447, Ala448, Leu482, Gln484	Leu99, Ala103,	Leu164, Arg166,	Val131, Ile133,
Pro232, Asn233, Gly234,		Val106, Met133,	Asp167, Ile168,	Asn269, Phe401,
Pro235, Trp236, Thr238,	SITE:2	Met134, Phe137,	Phe201, Tyr207,	Asp402, Pro409
Val239, Arg247, Pro290,	Gly56, Phe57, Cys58, Met59,	Ala159, Val162,	Ser208, Pro209,	
Arg296, Pro368.	Phe367, Tyr399, Leu475, Leu477,	Leu163, His166	Trp212, Tyr218,	SITE:3
	Leu479, Gly480, Leu482		Gly220, Ala224,	Trp215, Lus216,
SITE:3		SITE:3	Ser227	Val217, Phe219,
Asn233, Gly234, Pro235,	SITE:3	Leu4, Gly5, Tyr6,		Met244, Leu247,
Glu313, Val367, Val370,	Lys173, Ser312, Ser315, Phe316,	Arg17, Glu29,	SITE:3	Thr249, Leu264,
His405, Pro410, Gln413,	Gln484, Pro485, Lys487, Pro488,	Lys30, Lys31,	Phe100, Gly102,	Lys265, Leu309
Trp532, Asn533, Leu536,	Val489	Arg201,Leu203	Gln150, Val151,	
Pro537, Leu540			Ile173, Leu182,	SITE:4
	SITE:4	SITE:4	Leu184	Asp68, Cys231,
SITE:4	Leu142, Tyr347, Val350, Leu351,	Arg10, His14,		Met282, Pro395,
Arg475, Tyr479, Asn490,	Arg446, Leu449, Met450, Lys453	Tyr160, Asp161,	SITE:4	Lus413, Ala415,
Glu491, Asp494, Ala497,		Asp164, Met197	Phe130, Ile133,	Asn416
Pro498, Leu518.	SITE:5		Thr134, Asp234,	
	Asn159, Asp174, Ala178, Ser195,	SITE:5	Gly238, Asp239	SITE:5
SITE:5	Asn197, Asn198, Pro199, Gln200,	Leu110, Val113,		Phe56, Tyr59,
Glu81, Met85, Aspl31,	Asp201, Val204, Tyr307.	Tyr126, Phe169	SITE:5	Leu289, Leu397,
Val132, Thr436, Leu437,			Val96, Ser97,	Ile398, Met399,
Trp439, Tyr449, Glu452,			Ser98,Val103,	Ile411, Ala412
Ile457, Arg463, Tyr465			Ile104, Arg105	

P450: cytochrome P450, GST: gutathione S-transferase, PKC: protein kinase C, ACT: α-1-antichymotrypsin, AchE: acetylcholinesterase.

In the case of cytochrome P450n, the interaction energy in descending order with several varies in between -37.90 to -26.53 is -45.21, -43.97, -43.60, -37.23, -36.48, -34.94, -33.09, -31.42 and -25.21, respectively, for phosmet, azinphosmethyl, quinalphos, fenthion, chlorpyrifos, parathion, malathion, methylparathion, and monocrotophos and the corresponding number of H-bonds is 4, 3, 2, 1, 1, 4, 4, 3, and 2, respectively. The frequently involved amino acids in H-bonds are Arg105, Arg106, Arg212, Ala305, Thr310, Arg372, Glu374, Ile443, and Gly444 of human P450 protein (***Fig. 6B***). P450 isozymes have greater activity in direct detoxification of these above mentioned -45.21, -43.97, -43.60, -37.23, -36.48, -34.94, -33.09, -31.42 and -25.21 respectively; thus, it may be more important in assessing risk at low levels of exposure.

Similarly, in the case of GST, it was reported that GST was used for the detection of pesticides like atrazine. The nucleophilic attack of GST on atrazine releases the H^+^ ion, which can be detected as a pH change that directly correlates with the concentration of the analyte[11]. The binding energy in descending order with various organophosphorus pesticides is -39.60, -38.22, and -36.22 for azinphosmethyl, phosmet and malathion, respectively, and the lowest binding energy is -24.19 for tetrachlorvinphos. For interaction of GST with these pesticides, the frequent involvement of H-bonds varies between 1 and 4 and the highest interaction was found to be with monocrotophos (4) and their dock score is -26.15. Then, mostly hydrophobic residues involved in H-bonding are Trp7, Tyr6 and Tyr115. Some other amino acids that are involved in the interaction between GST and OPs are Arg42, Asn108 and Lys207.

**Table 3:** The global energy (dock score) of five different proteins i.e. acetylcholinesterase (AChE), cytochromeP450 (P450), glutathione S-transferases (GST), protein kinase C (PKC) and α-1-antichymotrypsin (ACT) with ten different organophosphorous pesticides

Ligands	Dock Score of AChE	NoofH-bond /Amino acids	Dock Score of P450	No of H-bond /Amino acids	Dock Score of GST	No of H-bond /Amino acids	Dock Score of PKC	No of H-bond /Amino acids	Dock Score of ACT	No of H-bond /Amino acids
Malathion	-34.30	4→Tyrl55,Ser234, Tyr368(2)	-33.09	4 → Pro107, Arg106(2), Arg372	-36.22	2→Asn108, Tyr115	-27.32	2→Arg73, Asp202	-23.75	2→Ser243(2)
Parathion	-37.90	5→Tyrl55, Tyrl03 (2), AsplOS, Thrl06	-34.94	4→Ala305, Thr310, Gly444, Ile443	-28.77	l→Asn108	-30.53	6 →Serl89, Ser75(2), Arg73 (3)	-28.04	2→GIn279 (2)
MethylParathion	-35.22	6→Trpl82(2), Argl3 (3), Asnl86	-31.42	3→ Gly444, He443, Argl05	-28.48	2 →Tyr115 (2)	-27.76	5 →Arg73, Asp 76(2), Met88(2)	-23.35	4→Serl34(2), Glnl32, Tyr210
Fenthion	-35.24	l→Tyrl33	-37.23	l→Arg212	-32.45	l→ Tyr115	-29.89	l→Ser75	-24.36	l→Lys413
Chlorpyrifos	-36.23	2→Tyr337(2)	-36.48	l→Arg212	-28.46	2→Trp7(2)	-32.98	3→Ser75(2), Glyl88	-26.70	l→Asp68
Monocrotophos	-26.53	4→Asp74, Tyr74, Tyrl24, Tlir75	-25.21	2→ Glul25, Ala289	-26.15	4→Trp7, Tyr6 (2), Tyr115	-21.91	4→Arg73, Phel87, Ser75(2)	-16.03	2→Asp68, Lys413
Quinalphos	-36.27	4→Glu81, Tyr465, Arg463(2)	-43.60	2→Gly481, Arg106	-30.66	2→Tyr6, Lys207	-29.67	3→Serl89, Asp202, Glyl88	-30.15	2→Glu245, Asp223
Phosmet	-36.23	2→Asn533,Arg417	-45.21	4→Arg130,Ile443 (2), Gly444	-38.22	l→Tyr6	-33.23	5 →Ser75(3), Arg73, Asp 76	-25.80	4→Thrl35(2), Glnl85 (2)
Tetrachlorvinphos	-30.71	4→Tyel24(2), Serl25, Tyr337	-26.85	2→ Serl09, Glu374	-24.19	3→Trp7, Arg42 (2)	-29.74	2 →Glu171, Aspl78	-23.15	2→Asp68, Gln283
Azinphosmethyl	-36.42	l→Asp306	-43.97	3→Glu374, Arg372 (2)	-39.60	2→Tyr115, Tyr6	-36.57	4 →Ser75 (2), Asp76 (2),	-25.63	4→Asnl25, Tyr208(2), Thr135

**Table 4:** The docking parameters used for bioinformatics analysis.

Identified protein's	Ligands with highest docked score	Global energy	Attractive Van der Wall force	Repulsive Van der Wall force	Atomic contact energy (ACE)
Acetylcholinesterase (Pdbid: 3LII)	Parathion	-37.90	-15.66	2.83	-10.50
Cytochrome protein P450 (Pdbid: 3NXU)	Phosmet	-45.21	-14.83	2.32	-15.72
Gutathione S-transferase (Pdbid: 4GTU)	Azinphosmethyl	-39.60	-15.23	3.46	-13.37
Protein kinase C (Pdbid: 2XJ1)	Azinphosmethyl	-36.57	-13.71	1.49	-11.03
Anti-chymotrypsin (Pdbid: 1YXA)	Quinalphos	-30.15	-14.07	0.77	-7.62

The highest interaction energy of all five proteins like acetylcholinesterase, cytochrome protein P450, gutathione S-transferase, protein kinase C and α-1-antichymotrypsin in H. sapiens with the top five different organophosphorous pesticides like parathion, phosmet, azinphosmethyl and quinalphos respectively that show the highest interaction with each other.

The fourth protein, PKC, is also the best target protein for organophosphorus pesticides because it can also affect signaling pathways by activating PKC[15]. However, this activation is probably indirect through organophosphorus pesticides-mediated formation of reactive oxygen species (ROS) that activate PKC. Organophosphorus pesticides that induced ROS formation and oxidative stress have also been shown to be associated with apoptosis in different tissues. It shows that organophosphorus pesticides can inhibit steroid androgen receptor, which can cause steroid hormone disturbances in the human body. The binding energy of PKC with organophosphorus pesticides in descending order is -36.57, -33.23, -32.98, -30.53 and -21.91, respectively, for azinphosmethyl, phosmet, chlorpyrifos, parathion, and monocrotophos (lowest) (***Fig. 5***). For interaction of PKC with the above mention pesticides, the frequent involvement of H-bonds varies between 1 and 6 and the highest interaction was found to be with parathion (6: Ser189, Ser75, and Arg73) followed by methylparathion (5: Arg73, Asp76, and Met88), phosmet (5: Ser75, Arg73, and Asp76) (***Fig. 6D***) that defined their toxicity with PKC.

In the case of α1-antichymotrypsin, which plays an important role in the digestive system, the interaction energy in descending order with various organophosphorus pesticides is -30.15, -28.04, -26.70, -25.63, and -16.03 for quinalphos, parathion, chlorpyrifos and monocrotophos, respectively (***Fig. 5***). The amino acids in α1-antichymotrypsin frequently involved in the formation of hydrogen bond interaction with methylparathion are Ser134, Gln132, and Tyr210, with azinphosmethyl, Asn125, Tyr208, and Thr135, and with phosmet, Thr135, Gln185, and Ser234, which indicates the interaction of organophosphorus pesticides with α1-antichymotrypsin (***Fig. 6E***) and organophosphorus pesticides could inhibit the activities of α1-antichymotrypsin. All the above mentioned five target proteins in *H. sapiens* that are part of the different metabolic and digestive pathways are assumed to bind with ten organophosphorus pesticides, which inhibit the enzymatic activity of these proteins and cause toxicity in the human body.

## DISCUSSION

The crystal structures showed good overall structural quality and were validated through PROCHECK, ERRAT plot and ProSA program, which revealed that PKC indicates a good quality model. Active sites and their involved residues are predicted through the Pocket finder and the CASTp server. In our docking study, the global energy function (i.e. dock score) of five proteins with their highest interaction energy with organophosphorus pesticides was calculated by the FireDock server to be -45.21 relative units. It is interesting to note that phosmet pesticides have shown a maximum dock score of -45.21 with P450 and the involved residues are Arg130, Ile443, and Gly444, which also involved in the active sites of the protein as shown in ***Fig. 6B***. Thus, our observation suggests that in the case of GST bound to azinphosmethyl pesticides, the highest dock score is -39.60 and the involved hydrophobic amino acids are Tyr115 and Tyr6. AchE bound to parathion shows that the third highest binding energy is -37.90 and the involved amino acid residues are Tyr155, Tyr103, Asp105, and Thr106, which also belongs to the active sites predicted through the pocket finder and the CASTp server as listed in ***Table 3*** and ***Fig. 4***. Similarly, the fourth and fifth highest binding energy was found between PKC protein and azinphosmethyl and between ACT and quinalphos, which is -36.57 and -30.15, respectively, and the number of H-bonding is 4 and 2, respectively. We analyzed protein-ligand interaction on five metabolic proteins interacting with ten organophosphorus pesticides and the aim of the study was to identify the most toxic organophosphorus pesticides. The docking results indicated that phosmet, azinphosmethyl, parathion and quinalphos, as the most toxic pesticides, inhibit the activity of enzymes and exert adverse effects on humans. Further analysis can be carried out in the wet lab to determine whether these findings are reflective of *in vivo* conditions.

In conclusion, the analysis of docking with ten organophosphorus pesticides with five target proteins (their PDBID are 3LII, 3NXU, 4GTU, 2XJ1 and 1YXA) in *H. sapiens* highlighted some important interactions operating at the molecular level and caused toxicity in the human body. The crystal structures were shown to possess good overall structural quality and were validated by using PROCHECK, ERRAT plot and the ProSA program. Active sites and their involved residues are predicted through the Pocket finder and the CASTp server. The most important aspect of the results is the interaction study in which the global energy is maximum at -45.21 for cytochrome P450 of *H. sapiens* with phosmet pesticides, which defines the more toxic nature of phosmet that exerts harmful effects on humans. AchE bound to parathion shows that the third highest binding energy is -37.90. The analysis of H-bonding in methylparathion with AchE, parathion with PKC and AchE, phosmet with PKC and methylparathion with PKC shows that the highest number of bond is 6, 6, 5, 5 and 5, respectively. Therefore, it is concluded that these organophosphorus pesticides are more toxic and inhibit the enzymatic activity by interrupting several metabolic and the digestive pathways in *H. sapiens*. Thus, this study will be useful for the prediction of the toxicity level of organophosphorus pesticides, herbicides and other toxic compounds, which inhibit the activity of enzymes. It provides guidance for further screening through experimental *in vitro* and *in vivo* analyses.
